# Assessment of Electrocardiogram Rhythms by GoogLeNet Deep Neural Network Architecture

**DOI:** 10.1155/2019/2826901

**Published:** 2019-04-28

**Authors:** Jeong-Hwan Kim, Seung-Yeon Seo, Chul-Gyu Song, Kyeong-Seop Kim

**Affiliations:** ^1^Biomedical Engineering, School of ICT Convergence Engineering, College of Science & Technology, Konkuk University, 268 Chungwon–daero, Chungju 27478, Republic of Korea; ^2^Division of Electronic Engineering, Chonbuk National University, 567 Baekje-daero, Jeonju 54896, Republic of Korea

## Abstract

The aim of this study is to design GoogLeNet deep neural network architecture by expanding the kernel size of the inception layer and combining the convolution layers to classify the electrocardiogram (ECG) beats into a normal sinus rhythm, premature ventricular contraction, atrial premature contraction, and right/left bundle branch block arrhythmia. Based on testing MIT-BIH arrhythmia benchmark databases, the scope of training/test ECG data was configured by covering at least three and seven *R*-peak features, and the proposed extended-GoogLeNet architecture can classify five distinct heartbeats; normal sinus rhythm (NSR), premature ventricular contraction (PVC), atrial premature contraction (APC), right bundle branch block (RBBB), and left bundle brunch block(LBBB), with an accuracy of 95.94%, an error rate of 4.06%, a maximum sensitivity of 96.9%, and a maximum positive predictive value of 95.7% for judging a normal or an abnormal beat with considering three ECG segments; an accuracy of 98.31%, a sensitivity of 88.75%, a specificity of 99.4%, and a positive predictive value of 94.4% for classifying APC from NSR, PVC, APC beats, whereas the error rate for misclassifying APC beat was relative low at 6.32%, compared with previous research efforts.

## 1. Introduction

Heart disease progresses because of insufficient blood supply to the heart when coronary artery disease develops or arrhythmias are severe and long-lasting [1]. According to statistics from the Centers for Disease Control and Prevention (CDC), heart disease is the world's leading cause of death. Consequently, early diagnosis of cardiovascular disease is important for reducing the devastating impact and increasing quality of life. The primary screening tool for heart disease, the electrocardiogram (ECG), provides diagnostic features that determine the existence of irregular heartbeats by measuring and recording the electrical activity of the heart [2].

Common heart-monitoring devices for cardiac arrhythmias include Holter equipment, which continuously collects 24 hours ECG data [3, 4]. Various ECG arrhythmia classification algorithms have been developed [5–8]. Artificial neural network models with backpropagation algorithms have also been proposed to classify ECG data into normal and abnormal patterns [9–11] by training and testing the PhysioNet MIT-BIH Arrhythmia benchmark database [12].

Recently, deep learning models trained on image data have been applied to interpret ECG data for automatic classification of arrhythmias [13–15]. Deep learning is a subfield of machine learning, and it aims to learn features from three or more hierarchical layers to solve the complex tasks that were difficult for shallow neural network models [16, 17].

Concerning patient-specific ECG heartbeat classification via a deep learning approach, Kiranyaz et al. [18] implemented a 1-dimensional convolutional neural network (CNN) classifier with training and testing MIT-BIH arrhythmia records acquired from 44 patients. This proposed CNN architecture achieved a classification performance detecting ventricular ectopic beats (VEBs) and supraventricular ectopic beats (SVEBs) with an accuracy of 99% and 97.6%, respectively. Zhang et al. [19] claimed a higher accuracy of detecting VEB and SVEB beats (99.7% and 99.3%, respectively) by proposing a patient-specific ECG classifier based on recurrent neural networks and a clustering technique. However, deep learning models for classifying ECG beats might encounter difficulties in overtraining caused by quasi-periodic behaviour of ECG data. Therefore, it is necessary to exploit the number of samples contained in an ECG segment for representing input variables to avoid overtraining. Thus, we first propose a way of determining the optimal number of ECG segments used to encode the input variables. Then, we build a modified deep neural network based on GoogLeNet deep learning architecture [20, 21] with modifying inception modules to classify ECG beats into premature ventricular contraction (PVC), atrial premature contraction (APC), right bundle branch block (RBBB), and left bundle brunch block (LBBB). For the experimental tests for ECG classification, the annotated information and raw data of the MIT-BIH database from 44 patients were evaluated by our proposed GoogLeNet deep neural architecture for expanding the kernel size of the inception structure.

## 2. Representations of ECG Segments

### 2.1. Determination of ECG Intervals for Encoding Input Data

To determine the number of ECG samples for supplying input data, we define *R*-peak features of ECG data as follows:

 
*R*_*n*_: reference beat in which the *n*^th^*R*-peak occurs in the training and test dataset
 
*R*_*n−k*_: time position in which the (*n* − *k*)^th^*R*-peak occurs with respect to the reference beat
 
*R*_*n+k*_: time position in which the (*n*+*k*)^th^*R*-peak occurs with respect to the reference beat
 
*R*_*n−k*_*·R*_*n+k*_: time interval between the time locations of *R*_*n−k*_-peak and *R*_*n+k*_-peak.

In our study, the range of training/test data, [*X*_*n*_^first^, *X*_*n*_^end^], ECG interval was configured to include *R*_*n*_*-*reference peaks by covering at least three and seven *R*-peaks prior and posterior to *R*_*n*_*-*peak by forming

(1) $$\begin{array}{c} X_{n}^{\text{first}}=R_{n-k+1}+\frac{R_{n}\cdot R_{n-k+2}}{2},\;k=3,4,5,6,7,\\ X_{n}^{\text{end}}=R_{n+1}+\frac{R_{n}\cdot R_{n+2}}{2}. \end{array}$$

**Figure 1:**
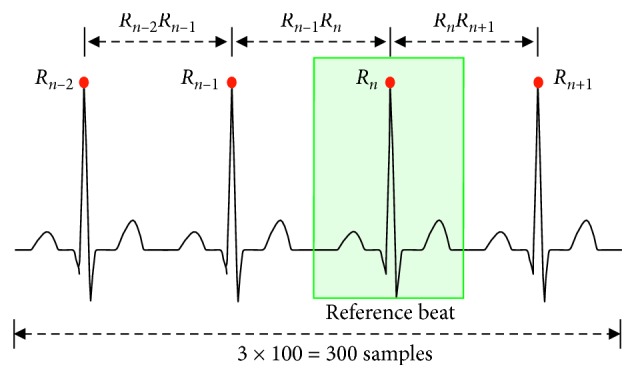
ECG segments normalization process for supplying input data.

Because the number of samples contained in the ECG interval was different depending on a patient's type of arrhythmia, a normalization process was necessary to unify the number of samples for training and testing input data. Kiranyaz et al. [18] and Zhang et al. [19] defined an ECG segment for the input data by including three *R*–*R* intervals. In our study, the test/training ECG data were acquired by performing the normalization process with multiplying the number of (*R*–*R* intervals-1) by 100 samples to avoid an aliasing problem.  Figure 1 shows an example of defining the data interval with four ECG segments and the number of normalized samples.

### 2.2. Training and Testing Set

To classify the ECG rhythm using our proposed deep learning architecture, the rhythm of arrhythmia was classified into normal sinus rhythm (NSR), APC, PVC, RBBB, and LBBB as input data for training and testing [22–24]. In the considered segment of the MIT-BIH arrhythmia database, various arrhythmias are present, and the classification is determined with a reference beat.

All 48 patients from the MIT-BIH arrhythmia data were randomly selected, excluding four with pacemakers.  Table 1 shows the total number of rhythms from the MIT-BIH arrhythmia data and the number of data used for training and testing the GoogLeNet deep learning model.

## 3. GoogLeNet Deep Learning Structure

The existing depth learning model can achieve high accuracy by deepening the layers to increase the performance of the neural network. A major drawback of this model is that the computational complexity increases exponentially as the layer becomes deeper. Google introduced the inception structure at 2014 ImageNet Large-Scale Visual Recognition Challenge (ILSVRC14), being the best-performing model and is called GoogLeNet [20]. At the core of this structure, the inner layer of the neural network was extended to output various correlation distributions based on the idea that the neural network output of each layer has optimal efficiency if various probability distributions with high correlations with the input data are obtained. In the basic inception v1 module, where input data are fed into four independent layers (1 × 1, 3 × 3, 5 × 5 convolution layers and 3 × 3 max-pooling layer), the outputs are combined into a single data set. Inside, the convolutional layers derive various spatial information of the input data, and the maximum pooling layer plays the role of extracting distinct features by reducing the channel and size of the input data. Therefore, the inception module is a method of extracting more information into a smaller layer by widening the layer of the neuron network, which is only composed of the existing depth.

### 3.1. Designing GoogLeNet Deep Learning Model for Arrhythmia Detection

**Figure 2:**
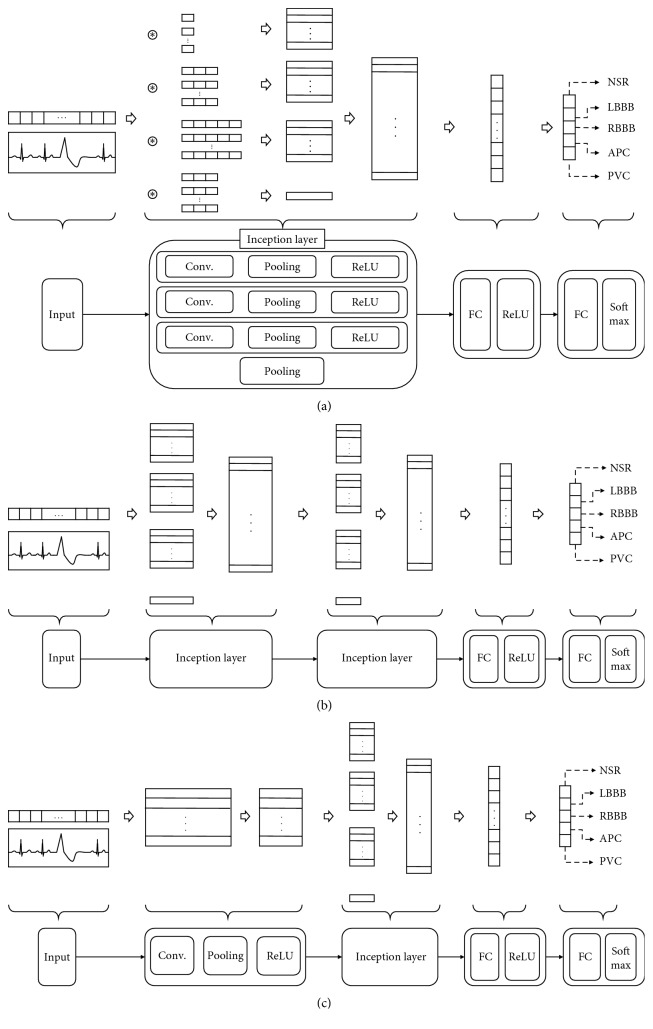
Modified GoogLeNet neural network architectures. (a) Design I: one inception layer. (b) Design II: two inception layers. (c) Design III: CNN layer + one inception layer.

Currently, the inception structure has been updated to v4. The shape of v1 is slightly extended. In this research, we use the v1 model as a basis to construct three CNN layers, an activation layer, and a maximum pooling layer.  Figure 2(a) shows a Design I model using a single inception module, consisting of a complete connection layer and an output layer, and Figure 2(b) represents a Design II model using two inception modules. In Figure 2(c), Szegedy et al. [20] claimed that the use of the incoherence structure was efficient after using the convolution layer.  Table 2 summarizes the composite specifications of the constructed incessant model and the detailed parameters for the pooling layer.

### 3.2. Optimization Parameters of the GoogLeNet Deep Learning Model

**Figure 3:**
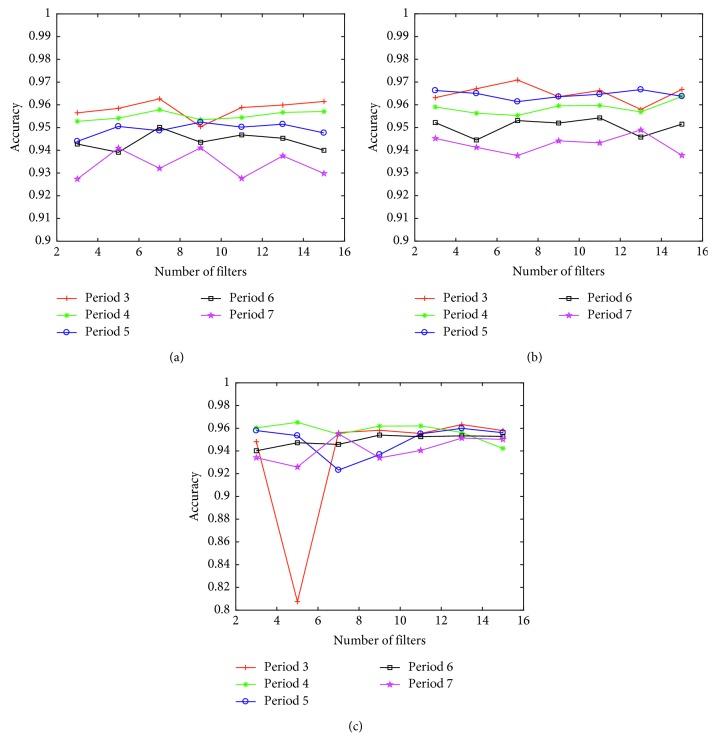
Accuracy of arrhythmia detection with varying the number of filters changed in the inception model. (a) Design I: one inception layer. (b) Design II: two inception layers. (c) Design III: CNN layer + one inception layer.

The proposed deep learning model was implemented by MATLAB codes using a desktop PC that comprised AMD FX-8350 CPU and 12 GB memory for matrix computational loads under the Windows 10 operating system. As the number of convolution filters inside the inception increases, the number of filters on the second floor increases by fixing the number of filters on the first floor of the reception to 15, when only the inception layer is used to confirm the change in the arrhythmia classification accuracy. We also evaluated the inception model using the convolution layer together. Even if the number of filters in an inception layer increases, as shown in Figure 3, the accuracy is not significantly enhanced. In fact, the accuracy of the model combined with the convolution layer, and inception layer is reduced by the difference in precision because of the ECG interval representation.

To ascertain the influence of the ECG segment on classification accuracy of the arrhythmia in our constructed inception model, the classification accuracy with the highest number of filters on each floor is summarized in Table 3. The combination model of the inception layer with the convolution layer reveals no clear difference from the model of the first floor of the reception, and the difference in accuracy of the ECG segment section decreased from 2.2% to 0.8%. The input data achieved by the inception layer was higher by about 1% in 2-layer inception models. The highest accuracy was achieved with three ECG segments.

### 3.3. Expansion of Kernel Size in the GoogLeNet Deep Neural Network Model

**Figure 4:**
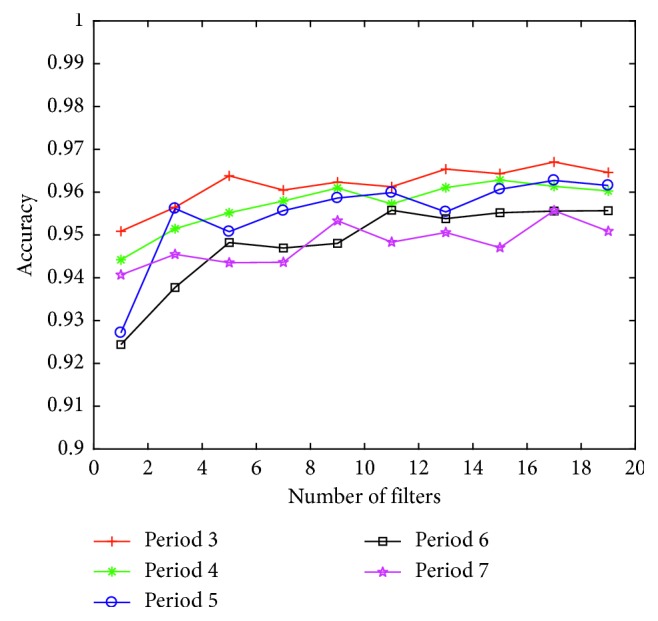
Accuracy of arrhythmia classification of as number of filters in expanded kernel model.

The conception filter of the basic inception module seems to be suitable for extracting the feature information of the normalized ECG with a kernel size of 1, 3, and 5, but may not be suitable for deriving information between *R*–*R* intervals. Therefore, by increasing the kernel size to 10, 50, and 100, time information included in the ECG such as the *R*–*R* interval of the ECG signal could be obtained. To apply this inception model, it can only be used in the first layer directly computed with ECG input data. The arrhythmia classification accuracy is summarized in Figure 4 and Table 4, showing the increasing number of internal convolution filters by 2 steps in the range from 1 to 19 of the inception layer. Thus, the number of filters increases, and the accuracy rises little by little, but the accuracy gets converged to a constant value from nine layers, and the maximum accuracy is achieved when it is comprised of three segments of ECG signals.

## 4. Results

### 4.1. Evaluation of Arrhythmia of Whole ECG Data

#### 4.1.1. Performance of Basic Inception Module

**Figure 5:**
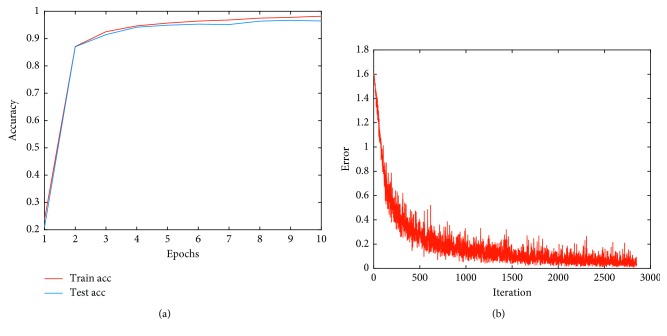
Training and testing result by basic inception module. (a) Accuracy. (b) Error.

The highest classification performance was achieved by considering three ECG segments.  Figure 5 shows the changes in accuracy and errors, and the arrhythmia classification index of the ECG signal is listed in Table 5. APC has the highest error rate of 13.99% of the percentage, and it costs a half portion of the total error at the rate judged to be an error of each arrhythmia rhythm. Therefore, we need to focus on reducing errors in APC.

#### 4.1.2. Expanded Inception Module

**Figure 6:**
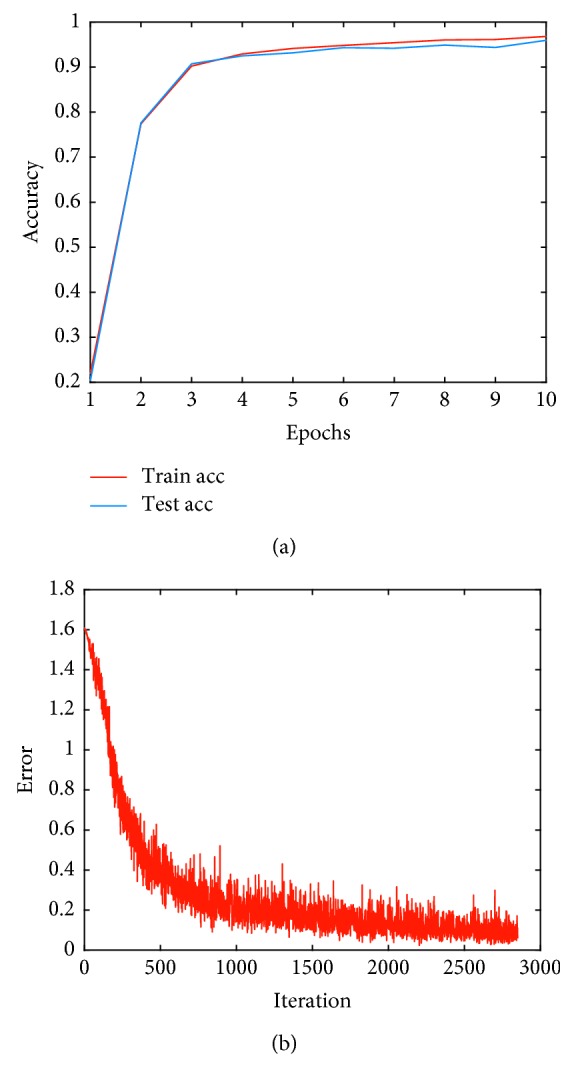
Training and testing of an inception module with expanding kernel size. (a) Sensitivity. (b) Error.

The highest accuracy parameter is obtained in the model in which the kernel size was expanded to the nine inception modules, and the number of input data and the number of filters in the ECG three segments are nine. When evaluating the ECG arrhythmia as shown in Figure 6, a change in accuracy and error appears as listed in Table 6, which shows the arrhythmia classification index. From the results of this model, the overall arrhythmia error increased; however, the error rate of APC decreased to 6.78%. Therefore, when using only the first filter on the first floor of inception and nine filters, we can be sure that it effectively responds to APC detection.

### 4.2. Evaluation of Patient-Specific Arrhythmia

The ECG signal can vary depending on the patient. The waveform of the cardiovascular symptoms such as the rhythm changes in shape depending on individual differences and the applied measurement device or method. In addition, when analyzing the rhythm of the MIT-BIH arrhythmia data, although two specialists aided in the evaluation, there were cases where the opinions were classified into rhythms that differ from each other. This is the reason why the type of arrhythmia differed for a given pattern. As a result of evaluating the MIT-BIH arrhythmia data with a deep learning model with five arrhythmia rhythms, the arrhythmia classification accuracy did not exceed 97%.

Therefore, we applied the deep learning model, which the evaluation rather presented the important rhythm of the individual custom heart, and classified the input data into the normal and the abnormal rhythm. Given that MIT-BIH 109, 111, 118, 124, 207, 214, and 232 NSR rhythm does not exist and only LBBB and RBBB rhythm exists, LBBB and RBBB can be regarded as normal rhythms. Furthermore, MIT-BIH arrhythmia data are about 2000 pieces for each number. The normal rhythms occupy a considerable part, the basic rhythms are about 20% of the verification data, and the abnormal rhythm is the number in total. Approximately 100 rhythms were sorted out, and the results were derived.

In our experimental simulations, the performance of accuracy, sensitivity, specificity, and positive predictive value (PPV) was evaluated, while varying the size of input data in our deep learning model during the training and test stage true positives (TPs) and true negatives (TNs) were used as metrics to detect abnormal heartbeats. TP refers to the judgements of arrhythmia rhythm, and TN defines the case of detecting NSR beats. Additionally, false positive (FP) refers to the decision of abnormal heart beat on NSR, and false negative (FN) defines the case of classifying NSR beats on the irregular heartbeats.

(2) $$\begin{array}{c} \text{Accuracy}\left(\text{Acc}\right)=\frac{\text{TP}+\text{TN}}{\text{TP}+\text{TN}+\text{FP}+\text{FN}},\\ \text{Sensitivity}\left(\text{Se}\right)=\frac{\text{TP}}{\text{TP}+\text{FN}},\\ \text{Specificity}\left(\text{Sp}\right)=\frac{\text{TN}}{\text{TN}+\text{FP}},\\ \text{PPV}\left(\text{Pp}\right)=\frac{\text{TP}}{\text{TP}+\text{FP}}. \end{array}$$

**Figure 7:**
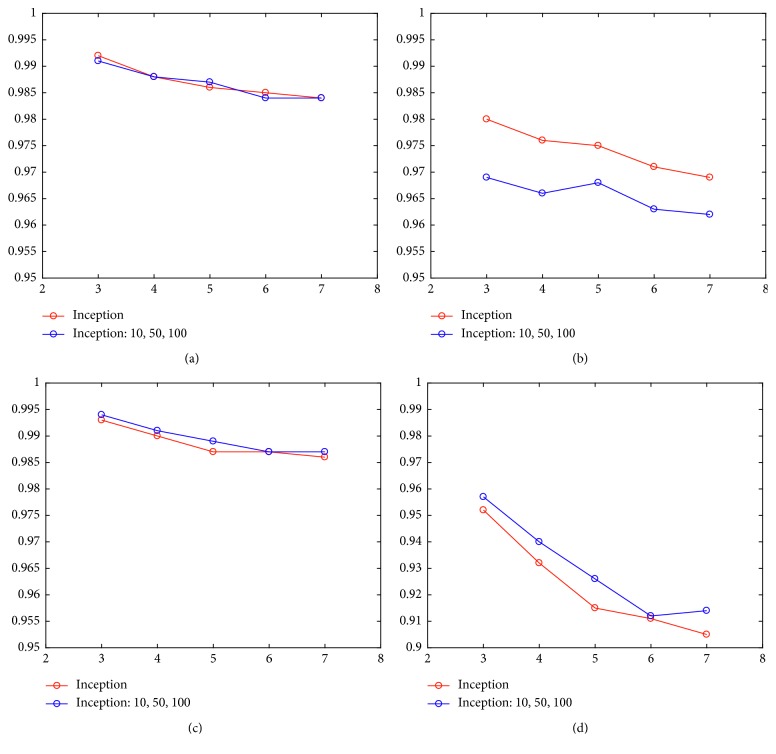
Performance index of patient-specific inception module. (a) Accuracy. (b) Sensitivity. (c) Specificity. (d) PPV.

Tables 7 and 8 list the accuracy, sensitivity, specificity, and PPV of all the considered data.  Figure 7 shows the comparison of results.

With regard to the accuracy and specificity with the four indicators, given that the NSR rhythms have more beats than the abnormal waveform in MIT-BIH database, a higher accuracy for the classification of NSR beats was obtained for all cases. Therefore, sensitivity and positive predictive value in the NSR classification must be judged more thoroughly. The sensitivity obtained by using an inception model with convolution filter sizes of [1, 3, 5] was between 96.2 and 96.9% accuracy, which is slightly higher than the expanded inception model. In contrast, in terms of positive prediction, the inception model with convolutional kernel size of [10, 50, 100] was somewhat higher with an accuracy ranging between 91.4 and 95.7%.

## 5. Conclusions

In this study, we explored the influence of detailed parameters by presenting a model suitable for the evaluation of ECG rhythm via various deep learning models. To accomplish this objective, the MIT-BIH ECG arrhythmia database was used to evaluate arrhythmia classification with varying of the inception structure to classify LBBB, RBBB, PVC, and APC rhythm. Based on Figure 4 and Table 4, the number of filters in the inception module should be at least 5 to detect arrhythmia beat. For the comparison with the previous state-of-the-art concerning the classification of heartbeats, we illustrated Table 9 to show the misclassification error rate of classifying NSR, APC, and PVC beats with the additional specifying accuracy, sensitivity, specificity, and positive predictive value in Table 10.

For the case of extended inception deep learning model used in our research, the misclassification error rate of APC was 7.2% for classifying NSR, LBBB, RBBB, APC, and PVC beats, whereas the error rate was 6.32% for classifying only NSR, APC, and PVC beats, which is relatively low compared with the previous research studies. Thus, we can conclude that the extension of the inception deep learning model can detect five distinct ECG rhythms with the highest accuracy of classification for the detection of APC beats.

## Figures and Tables

**Table 1:** Number of rhythms from MIT-BIH arrhythmia data used in our experiment.

	Total beats	Training beats	Test beats
NSR	71,446	10,000	5,000
LBBB	8,025	6,025	2,000
RBBB	7,128	5,728	1,400
APC	2,470	1,870	600
PVC	6,128	4,928	1,200

**Table 2:** Components of the deep learning model using GoogLeNet inception architecture.

Deep learning list	Parameters
Input size	200∼600
CNN layer	Number of filters: 15
	Kernel size: 5
	Stride: 1
	Padding: 0
Max-pooling layer	Pooling size: 2
	Stride: 2
	Padding: 0
Inception layer	Number of filters: 3∼15
	Kernel size: 1, 3, 5
	Pooling size: 3 × 3
	Stride: 1
	Padding: 0
FC layer	2 layers, [100, 50] neurons
Output size	5 classes
Iteration	10
Weight optimization function	Adam
Optimization parameters	Learning rate: 0.001, beta1: 0.9, beta2: 0.999
Batch size	100
Batch normalization	Not used
Dropout	Not used

**Table 3:** Mean accuracy of arrhythmia using the inception model.

	ECG segments (%)					Mean accuracy (%)
	3	4	5	6	7	
Design I	96.3	95.8	95.2	95	94.1	95.3
Design II	97.1	96.4	96.8	95.5	95.1	96.2
Design III	96.3	96.5	96	95.4	95.5	95.9
Mean accuracy (%)	96.6	96.2	96	95.3	94.9

**Table 4:** Accuracy of arrhythmia classification by the expanded kernel model with increasing number of filters.

Number of filters	ECG segments (%)					Mean accuracy (%)
	3	4	5	6	7	
1	95.1	94.4	92.7	92.4	94.1	93.7
3	95.6	95.1	95.6	93.8	94.5	94.9
5	96.4	95.5	95.1	94.8	94.4	95.2
7	96	95.8	95.6	94.7	94.4	95.3
9	96.2	96.1	95.9	94.8	95.3	95.7
11	96.1	95.7	96	95.6	94.8	95.7
13	96.5	96.1	95.5	95.4	95.1	95.7
15	96.4	96.3	96.1	95.5	94.7	95.8
17	96.7	96.1	96.3	95.6	95.6	96.0
19	96.5	96	96.2	95.6	95.1	95.9
Mean accuracy (%)	96.2	95.7	95.5	94.8	94.8	

**Table 5:** Arrhythmia classification using the basic inception module.

Ground truth	Classification result				
	NSR	LBBB	RBBB	APC	PVC
NSR	4,788	51	39	66	56
LBBB	19	1,973	3	3	2
RBBB	21	2	1,371	5	1
APC	41	3	6	541	9
PVC	10	6	1	14	1169
Misclassification error (%)	1.87	3.05	3.45	13.99	5.5

**Table 6:** Arrhythmia classification results of the inception module with increased kernel size.

Ground truth	Classification result				
	NSR	LBBB	RBBB	APC	PVC
NSR	4794	81	33	29	63
LBBB	26	1968	2	0	4
RBBB	25	3	1363	5	4
APC	62	14	15	489	20
PVC	19	4	1	4	1172
Misclassification error (%)	2.68	4.93	3.61	7.21	7.2

**Table 7:** Result of abnormal rhythm detection using the inception model.

Segment	Acc (%)	Se (%)	Sp (%)	Pp (%)
3	99.2	98	99.3	95.2
4	98.8	97.6	99	93.2
5	98.6	97.5	98.7	91.5
6	98.5	97.1	98.7	91.1
7	98.4	96.9	98.6	90.5

**Table 8:** Result of abnormal rhythm detection using the inception model with expanding kernel size.

Segment	Acc (%)	Se (%)	Sp (%)	Pp (%)
3	99.1	96.9	99.4	95.7
4	98.8	96.6	99.1	94.0
5	98.7	96.8	98.9	92.6
6	98.4	96.3	98.7	91.2
7	98.4	96.2	98.7	91.4

**Table 9:** Classification results compared to the state-of-the-art NSR, APC, and PVC heartbeats classification (%).

Truth	Classification result								
	Kiranyaz et al. [18]			Luo et al. [25]			Proposed		
	NSR	APC	PVC	NSR	APC	PVC	NSR	APC	PVC
NSR	73539	824	368	41873	300	947	4794	29	63
APC	837	1568	178	1520	282	9	62	489	20
PVC	230	72	5277	1240	13	1943	19	4	1172
Misclassification error (%)	1.43	36.36	9.38	6.18	52.61	32.98	1.66	6.32	6.61

**Table 10:** Classification results compared with the state-of-the-art PVC and APC heartbeats in terms of Acc, Se, Sp, and Pp (percentage, %).

Method	PVC				APC			
	Acc	Se	Sp	Pp	Acc	Se	Sp	Pp
Ince et al. [26]	98.3	84.6	98.7	87.4	97.4	63.5	99	53.7
Kiranyaz et al. [18]	99	93.9	98.9	90.6	97.6	60.3	99.2	63.5
Zhang et al. [19]	99.7	97.1	99.9	98.1	99.3	85.9	99.7	88.7
Luo et al. [25]	95.5	60.4	97.9	66.8	96.2	15.4	99.3	47.3
Proposed	98.64	98.40	98.70	94.90	98.31	88.75	99.40	94.40

## Data Availability

We tested PhysioNet MIT-BIH arrhythmia benchmark databases which are open public data available at http://www.physionet.org.
